# SARS-CoV-2 Among Military and Civilian Patients, Metro Manila, Philippines

**DOI:** 10.1093/milmed/usaa525

**Published:** 2020-11-30

**Authors:** John Mark Velasco, Fatima Claire Navarro, Paula Corazon Diones, Vicente Villa, Maria Theresa Valderama, Henry Tabinas, Domingo Chua, Romulo Dela Rosa, Melissa Monica Turao-Agoncillo, John Carlo Timbol, Susie Leonardia, Maria Leanor Timbol, Chonticha Klungthong, Piyawan Chinnawirotpisan, Khajohn Joonlasak, Wudtichai Manasatienkij, Angkana Huang, Anthony Jones, Stefan Fernandez

**Affiliations:** Department of Virology, U.S. Army Medical Directorate, Armed Forces Research Institute of Medical Sciences, Bangkok 10400, Thailand; National Institutes of Health, University of the Philippines Manila, Manila 1000, Philippines; V Luna Medical Center, Armed Forces of the Philippines Health Service Command, Quezon City 0840, Philippines; Department of Virology, U.S. Army Medical Directorate, Armed Forces Research Institute of Medical Sciences, Bangkok 10400, Thailand; V Luna Medical Center, Armed Forces of the Philippines Health Service Command, Quezon City 0840, Philippines; Department of Virology, U.S. Army Medical Directorate, Armed Forces Research Institute of Medical Sciences, Bangkok 10400, Thailand; V Luna Medical Center, Armed Forces of the Philippines Health Service Command, Quezon City 0840, Philippines; National Institutes of Health, University of the Philippines Manila, Manila 1000, Philippines; V Luna Medical Center, Armed Forces of the Philippines Health Service Command, Quezon City 0840, Philippines; V Luna Medical Center, Armed Forces of the Philippines Health Service Command, Quezon City 0840, Philippines; V Luna Medical Center, Armed Forces of the Philippines Health Service Command, Quezon City 0840, Philippines; Department of Virology, U.S. Army Medical Directorate, Armed Forces Research Institute of Medical Sciences, Bangkok 10400, Thailand; Department of Virology, U.S. Army Medical Directorate, Armed Forces Research Institute of Medical Sciences, Bangkok 10400, Thailand; Department of Virology, U.S. Army Medical Directorate, Armed Forces Research Institute of Medical Sciences, Bangkok 10400, Thailand; Department of Virology, U.S. Army Medical Directorate, Armed Forces Research Institute of Medical Sciences, Bangkok 10400, Thailand; Department of Virology, U.S. Army Medical Directorate, Armed Forces Research Institute of Medical Sciences, Bangkok 10400, Thailand; Department of Virology, U.S. Army Medical Directorate, Armed Forces Research Institute of Medical Sciences, Bangkok 10400, Thailand; Department of Virology, U.S. Army Medical Directorate, Armed Forces Research Institute of Medical Sciences, Bangkok 10400, Thailand; Department of Virology, U.S. Army Medical Directorate, Armed Forces Research Institute of Medical Sciences, Bangkok 10400, Thailand; Department of Virology, U.S. Army Medical Directorate, Armed Forces Research Institute of Medical Sciences, Bangkok 10400, Thailand

## Abstract

**Introduction:**

Severe acute respiratory syndrome coronavirus 2 (SARS-CoV-2) belonging to the family *Coronaviridae* and genus *Betacoronavirus* is the causative agent of COVID-19 disease and was first identified in Wuhan, China. SARS-CoV-2 spread globally with >28 million cases and 911,000 deaths recorded worldwide as of September 12, 2020. The Philippines reported the first case of community transmission on March 5, 2020, and despite the government imposing one of the longest and strictest lockdowns in Southeast Asia, the number of confirmed COVID-19 cases still surged with >250,000 cases and 4,000 deaths reported as of September 12, 2020. It is important to estimate the burden and impact of SARS-CoV-2 on the military population since this can affect the military readiness.

**Materials and Methods:**

Nasopharyngeal and oropharyngeal swabs were collected and SARS-CoV-2 real-time RT-PCR testing was performed on the samples. Phylogenetic analysis was performed using sequences from 23 SARS-CoV-2-positive specimens from this study and sequences retrieved from GenBank and GISAID databases.

**Results:**

From April 14 to August 15, 2020, a total of 12,432 specimens were tested with 763 (6%) unique individuals testing positive for SARS-CoV-2 by rRT-PCR. In the military population, majority of the patients who were tested (80%) and those who tested positive for SARS-CoV-2 (86%) were male. Military and civilian status was available for 7,672 patients with 515/5,042 (10%) positive among military patients and 248/2,630 (9%) positive among civilian patients. Both military and civilian populations had the highest case counts of SARS-CoV-2-positive cases in the 21- to 30- and 31- to 40-year-old age groups, while the 71- to 80-year-old age group had the highest proportion (18%) of SARS-CoV-2-positive cases. Sequencing analysis showed 19 different variants in the 23 genomes. Twenty of the 23 genomes were classified under clade GR/B1.1, 2 genomes were classified under clade GR/B1.1.28, and 1 genome was classified under Clade O/B.6. Twenty-two of the 23 sequences collected after June 25, 2020, contained the D614G mutation.

**Conclusion:**

We describe here the results of SARS-CoV-2 testing for military and civilian patients and personnel. The 21- to 30- and 31- to 40-year-old age groups had the highest case counts of SARS-CoV-2-positive cases. Sequencing results showed the presence of the D614G mutation in the spike protein in a majority of specimens collected from the end of June to July 2020.

## BACKGROUND

Severe acute respiratory syndrome coronavirus 2 (SARS-CoV-2) belonging to the family *Coronaviridae* and genus *Betacoronavirus* is the causative agent of COVID-19 disease and was first identified in Wuhan, China.^1^ SARS-CoV-2 spread globally with >28 million cases and 911,000 deaths recorded worldwide as of September 12, 2020.^2^ In the Philippines, the first confirmed SARS-CoV-2 case was a Chinese tourist from Wuhan, traveling in Bohol, Philippines, and was reported on January 30, 2020,^3^ the same day that the World Health Organization Director-General declared SARS-Cov-2 as a public health emergency of international concern.^2^ The Philippines reported the first case of community transmission on March 5, 2020, and despite the government imposing one of the longest and strictest lockdowns in Southeast Asia, the number of confirmed COVID-19 cases still surged with >250,000 cases and 4,000 deaths reported as of September 12, 2020.^2^

It is important to estimate the burden and impact of SARS-CoV-2 on the military population since this can affect the military readiness and because the unique factors associated with the military such as close-quarter living conditions and deployment to high SARS-CoV-2 transmission areas to assist in COVID-19 control efforts may put them at a higher risk for acquiring the infection. The Philippine military is also playing a major role in providing support to the civilian sector through various capacities (i.e., manning of triage and swabbing facilities, deployment to augment healthcare workers in areas with surges in COVID-19 cases, and logistical support in shipping supplies to various parts of the country). Though previous reports have described COVID-19 outbreaks in military forces, there is a need to know whether there are differences in SARS-CoV-2 prevalence and distribution in the Philippine military versus Philippine civilian populations.

In April 2020, through the guidance and efforts of the Philippines-Armed Forces Research Institute of Medical Sciences (AFRIMS) Virology Research Unit, Manila (PAVRU-M) under the Virology Department of the U.S. Army Medical Directorate Armed Forces Research Institute of Medical Sciences (USAMD-AFRIMS), Bangkok, Thailand, and in cooperation with the Armed Forces of the Philippines (AFP) military medical command of the V Luna Medical Center (VLMC), Armed Forces of the Philippines Health Service Command (AFPHSC), Quezon City, testing for SARS-CoV-2 was initiated at the AFP-AFRIMS Collaborative Laboratory.

## METHODS

### Collection and Testing Sites

V Luna Medical Center is a tertiary care, teaching hospital under the Armed Forces of the Philippines Health Service Command (VLMC-AFPHSC), located in highly urbanized Quezon City, National Capital Region, Philippines. This hospital is the largest tri-service hospital of the of the AFP and serves active duty military personnel, their dependents, and authorized civilians. VLMC served as a collection site and the central administrative center for specimens collected from other military hospitals and civilian swabbing facilities being manned by military personnel. All swabbing facilities were located in urban areas of Metro Manila. Sample testing was done at the AFP-AFRIMS Collaborative Molecular Laboratory and the VLMC Molecular Laboratory.

### Sample Collection

Nasopharyngeal and oropharyngeal swabs were collected using Dacron-tipped swabs by personnel at the VLMC triage area and from other military hospitals and civilian swabbing facilities all located within Metro Manila. Samples were collected from military and civilian persons under investigation for COVID-19, patients seeking clinical care and showing signs of COVID-19-like illness, or asymptomatic patients as part of contact tracing procedures. Swabs were stabilized in various Universal Transport Media (Copan, CA, USA; Sansure Biotech, Hunan, China; and Sanli, Liuyang, China), temporarily stored at 4°C, transferred to freezers (−80 ± 20°C), and tested within 24 to 72 hours.

### SARS-CoV-2 Real-Time RT-PCR Testing

Nasopharyngeal and oropharyngeal swabs were collected by VLMC triage personnel and rRT-PCR testing was performed by a combination of AFRIMS and VLMC laboratory personnel. The swabs were heat inactivated at 65°C for 10 minutes and viral RNA was extracted using the NATCH CS automated nucleic acid extraction machine (Sansure Biotech, Hunan, China) or manual RNA extraction kits: QIAmp viral RNA mini kit (Qiagen, Germantown, MD, USA), Sansure RNA one-step nucleic acid release reagent (Sansure, Biotech, Hunan, China), and GenAmplify RNA extraction kit (Manila HealthTek, Manila, Philippines) following manufacturers’ instructions. Briefly, specimens were thawed and an aliquot of 200 µL (Sansure) or 140 µL (QIAmp and GenAmplify) of each specimen was used for RNA extraction and eluted in 50 µL following manufacturer’s instructions of each kit. The following SARS-CoV-2 rRT-PCR kits were used following testing conditions and Ct cutoff values indicated by each manufacturer: Sansure (Sansure Biotech, Hunan, China), 40 Ct; BGI Genomics (Shenzhen, China), 37 Ct; modified Berlin assay, 38 Ct; and modified U.S. Army Medical Research Institutes of Infectious Diseases-Centers for Disease Control and Prevention (USAMRIID-CDC, USA) assay, 38 Ct. The extracted nucleic acid was amplified using the following PCR machines: ABI 7500 (Applied Biosystems, CA, USA), QuantStudio 7 (Applied Biosystems, CA, USA), SLAN96P (Sansure Biotech, Hunan, China), and MA6000 (Sansure Biotech, Hunan, China).

### SARS-CoV-2 Genome Sequencing and Phylogenetic Analysis

SAR-CoV-2 genome from 23 positive specimens were sequenced at USAMD-AFRIMS, Bangkok, Thailand, using a MiSeq (Illumina, USA), the results of which were reported and described previously.^4^ Phylogenetic analysis was performed using sequences from this study and sequences retrieved from GenBank and GISAID databases on August 24, 2020. The tree was generated using IQ-tree v. 1.6.12 using the generalized time-reversible (GTR + F + I) model and 1,000 bootstrap replicates.^5^ Multiple sequence alignments were performed using “Multiple Alignment using Fast Fourier Transform” v7.407 with the default setting.^6^ The phylogenetic tree was drawn with FigTree v1.4.4^7^ and lineage was determined using Pangolin v.2.0.4 (github.com/cov-lineages/pangolin),^8^ shown in Fig. 2, including the lineage and the clade of the 23 coding-complete genome sequences from this study. The genome comparisons to the Wuhan-Hu-1 reference genome (GenBank accession number NC_045512.2) were visualized using BRIG v0.95.^9^

### Statistical Analysis

The means (for quantitative variables) and proportions (for categorical variables) were calculated to describe the distribution of SARS-CoV-2-positive cases per age group. The associations among categorical variables were estimated and tested for significance using the VassarStats 2 × 2 contingency table using chi-square test of association or Fisher’s exact probability test, where appropriate (http://vassarstats.net/odds2x2.html; accessed October 2020). Two-tailed *P* values <.05 were considered statistically significant.

### Ethical Approval

Informed consent was obtained from the patients for SARS-CoV-2 rRT-PCR testing and sequencing. Sample collection was considered as a public health effort and did not require an Institutional Review Board–approved human use protocol.

## RESULTS

**FIGURE 1.**
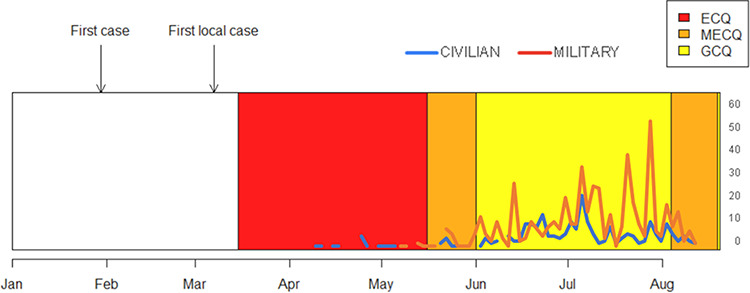
SARS-CoV-2 cases in military and civilian population per day (*n* = 763). ECQ—enhanced community quarantine (no movement regardless of age and health status: minimal economic activity except for utility service and critical economic sector, no transportation activity, and suspension of physical classes). MECQ—modified enhanced community quarantine (limited movement within ECQ zone for obtaining limited essential services and works: operation of selected manufacturing and processing plants up to 50% workforce, limited transporting services for essential goods and services, and suspension of physical classes). GCQ—general community quarantine (limited movement to services and work within GCQ zone; operation of government officers and industries up to 75% of workforce, limited transporting services to support government and private operations; flexible learning arrangements; and operate at limited capacites to cater to students).

From April 14 to August 15, 2020, a total of 12,432 specimens were tested with 763 (6%) unique individuals testing positive for SARS-CoV-2 by rRT-PCR. The majority were males (65%) in the overall tested population. Fig. 1 shows the SARS-CoV-2 rRT-PCR positive military and civilian cases detected from the start of testing in April to mid-August 2020 as well as the corresponding quarantine measures and duration of implementation with enhanced community quarantine (ECQ) being the most strict and general community quarantine (GCQ) being the least strict in terms of movement restriction.

The average number of specimens tested daily from April to June ranged from 32 to 51 samples with a maximum of 134 samples tested in 1 day. Daily testing increased to an average of 167 and 205 samples per day for the month of July and August, respectively, with 447 samples as the highest number of samples tested in 1 day.

The SARS-CoV-2 positivity rate of samples tested from April through June ranged from 1% to 2% and in July the positivity rate significantly increased to 9% (*P* value < .0001) with the positivity rate further increasing to 11% for August 1 to 15, 2020. Military and civilian status was available for 7,680 patients with 515/5,046 (10%) positive among military patients and 248/2,634 (9%) positive among civilian patients. Military and civilian status was not available for 4.752 patients. Among those who tested positive for SARS-CoV-2 by real-time PCR, 515 (67%) were military personnel and 248 (33%) were civilians. In the military population, the majority of the patients who were tested (80%) and those who tested positive for SARS-CoV-2 (86%) were male.

Table I shows the distribution of SARS-CoV-2-positive cases according to age group and military or civilian classification with 515/763 (67%) active duty military personnel comprising the majority of positive cases. Both military and civilian populations had the highest case counts of SARS-CoV-2-positive cases in the 21 to 30 and 31 to 40 years old age groups, while the 71 to 80 years old age group had the highest proportion (18%) of SARS-CoV-2-positive cases.

**TABLE I.** Military and Civilian (MILCIV) Distribution of SARS-CoV-2 Cases According to Age Group

	Military		Civilian					Overall				
Age group	SARS- CoV- 2 pos (*n* = 515)	(%)	SARS- CoV- 2 pos (*n* = 248)	(%)	Odds ratio	[95% CI]	*P* value	Total SARS- CoV- 2 pos (*n* = 763)	(%)	No MILCIV status; SARS- CoV-2 neg (*n* = 4,760)	Total SARS- CoV- 2 neg (*n* = 11,669)	Total (*N* = 12,432)
0-5	—	—	8/195	(4)	—	—	—	8/195	(4)	—	187	195
6-10	—	—	5/102	(5)	—	—	—	5/102	(5)	—	97	102
11-20	3/28^a^	(11)	12/235	(5)	2.23	[0.59-8.44]	.38	15/543	(3)	280	528	543
21-30	164/1,580	(10)	49/403	(12)	0.84	[0.60-1.18]	.35	213/3,566	(6)	1,583	3,353	3,566
31-40	179/1,877	(10)	48/422	(11)	0.82	[0.59-1.15]	.29	227/3,749	(6)	1,450	3,522	3,749
41-50	136/1,208	(11)	32/347	(9)	1.25	[0.83-1.87]	.33	168/2,571	(7)	1,016	2,403	2,571
51-60	33/349^b^	(9)	41/357	(11)	0.8	[0.50-1.31]	.45	74/1,137	(7)	431	1,063	1,137
61-70	—	—	31/431	(7)	—	—	—	31/431	(7)	—	400	431
71-80	—	—	20/109	(18)	—	—	—	20/109	(18)	—	89	109
81-90	—	—	2/29	(7)	—	—	—	2/29	(7)	—	27	29
Total	515/5,042	(10)	248/2,630	(9)	—	—	—	763/12,432	(6)	4,760	11,669	12,432

—, active duty military age: 18 to 56 years.

a Includes 18- to 20-year-old age group for the active duty military population.

b Includes 51- to 56-year-old age group for the active duty military population.

**FIGURE 2.**
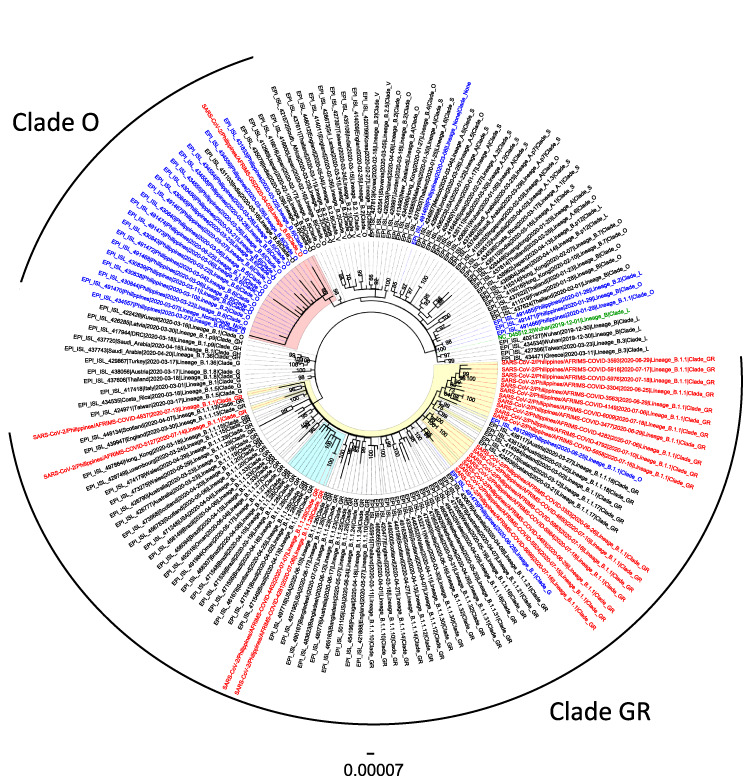
Maximum likelihood phylogenetic tree of 158 sequences from SARS-CoV-2 coding sequences (29,409 bp) including 23 sequences from the Philippines collected from April 3, 2020 to July 18, 2020 (red), 135 sequences from GenBank and GISAID databases (113 from other countries in black, 22 from the Philippines in blue).

Figure 2 shows the maximum likelihood phylogenetic tree of 161 coding sequences, which included 23 whole genome sequences from SARS-CoV-2-positive specimens from this study collected between April 3 to July 18, 2020, and 138 SARS-CoV-2 sequences from GenBank and GISAID databases (25 sequences from Philippines and 113 from other countries). We detected 19 different variants in the 23 genomes. Four amino acid substitutions (ORF1b-P314L, S-D614G, N-R203K, and N-G204R), unique to clade GR (both lineages B.1.1 and B.1.1.28), were seen in all sequences in this clade. Twenty of the 23 genomes were classified under clade GR/B1.1, 2 genomes were classified under clade GR/B1.1.28, and 1 genome was classified under Clade O/B.6. Twenty-two of the 23 sequences collected after June 25, 2020, contained the D614G mutation.

## DISCUSSION

The prevalence for SARS-CoV-2 for both military and civilian populations in this study were similar at 10% and 9%, respectively. Though the majority of those who tested positive for SARS-CoV-2 in this study were active duty military personnel (67%) it should be kept in mind that the collection sites were mainly catering to the military and not the civilian population. Patients who tested positive for SARS-CoV-2 had more complete data available since these were required by the Department of Health for tracking and quarantine purposes but for those who tested negative for SARS-CoV-2, a considerable number had incomplete data (ie, military or civilian status) and non-collection of data was due to extraordinary limitations in manpower, logistics, and high patient volume brought about by the pandemic.

The 21 to 30 and 31 to 40 years old age groups for both military and civilian populations had the highest SARS-CoV-2-positive case counts and is reflected in the peak distribution of COVID-19 cases based on the national data (https://endcov.ph/dashboard/; accessed October 28, 2020) and a statement by the World Health Organization Western Pacific Regional Office that the driving force of the coronavirus pandemic is coming from the working sector, namely the 20 to 40 year old age group with more than half of the COVID-19 cases in the Philippines belonging to these age groups (WPRO virtual press release, August 18, 2020).

Multiple introduction of various lineages, particularly clade GR/lineage B.1.1, a major European lineage most frequently found in Europe and exported worldwide^10,11^ was seen in 20/23 genomes from specimens collected between June 25 and July 18, 2020. Two genomes from specimens collected from July 6 and July 7, 2020, were classified under clade GR/lineage B.1.1.28, found in Brazil (86%), the UK (10%), and Australia (2%) (https://cov-lineages.org/lineages/lineage_B.1.1.28.html), while a single genome was classified under clade O/lineage B.6, a global lineage mostly found in India (43%), Singapore (40%), and Australia (5%) (https://cov-lineages.org/lineages/lineage_B.6.html).

These various lineages could have been introduced by Filipino repatriates coming from all over the world and who numbered >164,000 as of September 6, 2020 (Philippine Department of Foreign Affairs, unpublished data). It is important to determine the interactions and predominance of these different SARS-CoV-2 strains coming from different parts of the world and which may be introduced by travelers or by repatriates, despite restrictions in air travel. Particular strains may present with slightly different clinical presentations^12^ or transmission efficiency,^13^ which subsequently may have an impact on the local epidemiology of SARS-CoV-2, patient management, and necessary control measures. We detected the D614G mutation from SARS-CoV-2-positive specimens collected from the end of June to July in the majority (22/23) of the strains we sequenced. We subsequently observed a rapid rise of positive SARS-CoV-2 cases at VLMC starting July, and this observation was also reflected at the national level. The D614G mutation has been associated with higher titers of SARS-CoV-2 pseudo viruses (a retrovirus that can integrate the envelope glycoprotein of another virus to form a virus with an exogenous viral envelope with the genome retaining the characteristics of the original retrovirus) and higher viral RNA levels *in vitro*^13^ as well as enhanced SARS-CoV-2 replication in the upper airway using a primary human airway tissue model,^14^ though further studies are still needed to definitely determine whether this mutation translates to an actual impact on transmission efficiency^15^ or disease severity. It should be noted that movement restrictions were downgraded to GCQ at the National Capital Region starting from June, and the more relaxed quarantine measures could have been the main driving force for the increase in the number of cases. Alternatively, this might have also facilitated the dissemination of strains such as GR/B.1.1 and GR/B1.1.28 with the D614G mutation, which led to the replacement of other circulating SARS-CoV-2 strains before June 2020.

## CONCLUSIONS

We describe here the results of SARS-CoV-2 testing for military and civilian patients and personnel. The 21 to 40 years old age groups had the highest case counts of SARS-CoV-2 infection recorded, while the 71 to 80 years old age group had the highest proportion of SARS-CoV-2 positives among all the age groups. Sequencing results showed the presence of the D614G mutation in the spike protein in the majority of specimens collected from the end of June to July 2020, and this finding was associated with a sharp increase of SARS-CoV-2 cases seen at VLMC and in the entire Philippines starting July 2020. In this study, we found the D614G mutation in the majority of the sequences from specimens collected from the end of June 2020. The presence of this particular mutation may partially explain the rapid rise of cases in the Philippines in addition to the relaxation of quarantine measures. Strains with this particular mutation may be replacing the original SARS-CoV-2 strain/s circulating before June 2020.

This study had several limitations. Clinical outcome data were not available, and we were not able to assess the severity of disease presentation or determine the asymptomatic proportion among those who tested positive. A substantial number of patients had no military or civilian status, thus ascertaining the prevalence of SARS-CoV-2 in both populations cannot be performed accurately. Although the collection site is the largest tertiary military hospital of the AFP, the data we present might still be an underestimation of the prevalence and distribution of SARS-CoV-2 in the military population.

We recommend that targeted population (ie, healthcare workers) serologic cohort studies be conducted to clarify the infectious disease dynamics of SARS-CoV-2 infection. This approach can provide a more accurate estimate of disease burden, track age-specific infection rates, and provide information on the proportion of certain populations (ie, military) and age groups who have immunity. Obtaining this information is essential for targeted vaccination efforts and vaccine prioritization once a licensed vaccine is available. Sequencing of additional samples is also critical to track the different circulating strains in other geographical parts of the country and to correlate this with disease severity or efficiency of SARS-CoV-2 transmission.

Finally, this underscores the strong partnership maintained by the AFP medical command and USAMD-AFRIMS since the 2009 H1N1 pandemic, during which PAVRU-M supported disease testing within the AFP military healthcare setting. With the onset of the COVD-19 pandemic, this strong collaboration is once again highlighted as PAVRU-M and the AFP medical command work together in the fight against SARS-CoV-2.

## Data Availability

The SARS-CoV-2 genomes from the Philippines were a deposited in the GenBank database (accession nos. MT919768-90). The raw reads have been deposited in the NCBI Sequence Read Archive (SRA accession nos. SRS7273360-82). The Bio Project accession no. is PRJNA659293. The Bio Sample accession nos. are SAMN15903138-60.
